# The Potential of Neonatal Organ Donation in Central Sweden

**DOI:** 10.1177/09636897241303269

**Published:** 2025-01-24

**Authors:** Emil Bluhme, Ewa Henckel, Boubou Hallberg, Carl Jorns

**Affiliations:** 1 Department of Clinical Science, Intervention and Technology, Karolinska Institutet, Stockholm, Sweden; 2 Department of Transplantation, Karolinska University Hospital, Stockholm, Sweden; 3 Department of Neonatology, Astrid Lindgren Children’s Hospital, Karolinska University Hospital, Stockholm, Sweden

**Keywords:** organ donation, transplantation, neonate

## Abstract

Pediatric organ transplant recipients have a higher risk for wait list mortality due to the scarcity of size matched organs. Neonatal organ donation could potentially ameliorate the discrepancy but is currently not implemented in Sweden. This study aims to evaluate the potential of neonatal organ donation in central Sweden using a standardized protocol with organ specific criteria. Data on 2,061 neonates who deceased in central Sweden from 2006 to 2016 were collected; 308 neonates met criteria for possible donation. Medical records of all possible donors were reviewed, identifying 85 potential donors. Main cause of death was hypoxic ischemic encephalopathy 47% (n = 40). Median weight was 2,355 (IQR: 1,953) g, with 31% receiving inotropic support. Median creatinine of 72 (IQR: 67) µmol/l, urine production 3 (IQR: 2.2) ml/kg/h, ALT 0.51 (IQR: 1.5) µkat/l, and AST 1.7 (IQR: 3.1) µkat/l. Criteria for kidney donation was met in 39 potential neonatal, 29 for liver and 18 for heart, corresponding to a potential increase of 1.9, 1.4, and 0.9 donors PMP per year, respectively. In total, 16 neonates had a catastrophic neurological injury in combination with lack of brainstem reflexes, indicating plausibility of donation after brain death. Expanding organ donation into the neonatal period in Sweden could lead to an increase in organs available for transplant.

## Graphical abstract

**Graphical Abstract:**
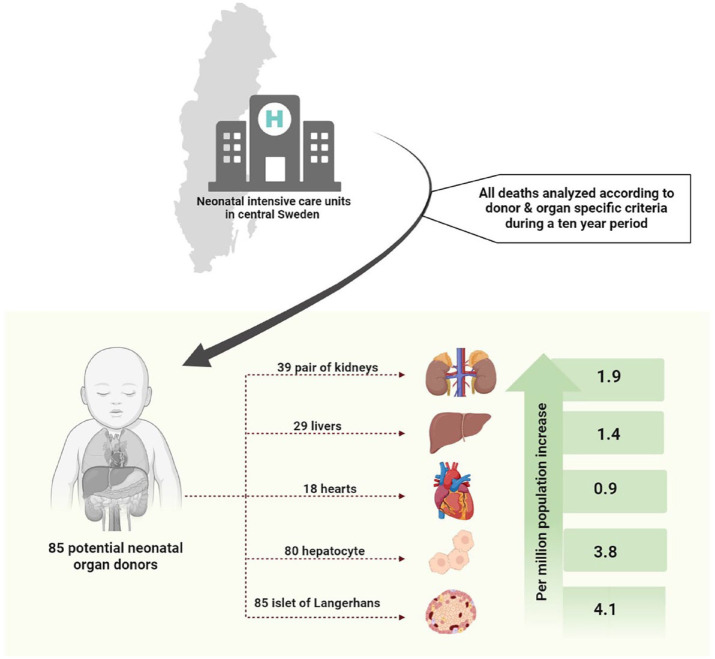

## Introduction

Transplantation of heart, lung, liver, and intestine provides life-saving procedures in the treatment of patients with terminal organ failure. Transplantation of kidneys and pancreas improves patient survival and quality-of-life compared with other available treatments. Cell therapies such as transplantation of Islets of Langerhans and hepatocytes are new treatment modalities in which clinical outcome has continuously improved over recent years^1,2^. However, both transplantation modalities are dependent on organ donors and the demand outreaches the supply. Still today, a considerable percentage of patients die on the waiting list.

Infants and other pediatric recipients are at a significantly higher risk of mortality on the waiting list compared with other age groups reaching up to 31% for heart and more than 10% for liver recipients^3,4^. The main reason for this excess mortality is a scarcity of size matched donors^5,6^. Furthermore, only organs from donors that are considered unsuitable for solid organ transplantation are available for cell isolation purposes. Hence, progress in the field of cell transplantation is hampered partly by the lack of access to donor tissue of sufficient quality.

Expanding organ donation into the neonatal period could aid in addressing these imbalances, both as a source for solid organ transplantation, as well as for cell therapies. Furthermore, donor organs from neonates could permit size matched transplantations for smaller recipients on the waiting list^7^. Although Sweden has among the lowest childhood mortality, most pediatric deaths occur in the neonatal period^8,9^. Indicating that neonatal organ donation could significantly increase the organ pool.

Cases of successful heart, lung, liver, multivisceral, and kidney transplantation from neonatal donors have been reported^7^. Furthermore, hepatocytes isolated from neonatal donors seem to be of superior quality in terms of yield, viability, and function compared with adult hepatocytes^10,11^. Successful cases of clinical hepatocyte transplantation using neonatal liver cells have been performed in four patients with urea cycle disorders, as well as a bridging treatment toward liver transplantation^12,13^.

Evidence suggest that neonatal organs are suitable for both solid organ transplantation and hepatocyte transplantation^7^. Yet, neonatal organ donation is currently not considered in Sweden. A neonatal donation program could potentially reduce mortality and waiting time for patients on the transplant waiting list. The aim of this study is to analyze the potential of a neonatal donation program with specified organ donation criteria in the central region of Sweden, and to assess its potential contribution to the current donor pool.

## Methods

Study inclusion was based on The Swedish neonatal quality registry (SNQ). SNQ covers all of Sweden’s 37 neonatology wards including all infants admitted for neonatal care from birth until 28 days postpartum, with a completeness of 98%–99%^14^. Possible and potential donor definitions were based on previous published definition by Domínguez-Gil et al.^15^; A possible deceased organ donor is defined as a patient with brain injury or lesion or a patient with circulatory failure and apparently medically suitable for organ donation. Potential donor can be categorized into a potential DCD or DBD donor. Potential DCD donor is defined as; a person whose circulatory and respiratory functions have ceased, and resuscitative measures are not to be attempted or continued. Or a person in whom cessation of circulatory and respiratory functions is anticipated to occur within a time frame that will enable organ recovery. Conversely, a potential DBD donor is defined as a person whose clinical condition is suspected to fulfill brain death criteria.

### Patient Cohort

Data was extracted on all deceased patients admitted to a neonatal intensive care unit (NICU) in Sweden during 2006–2016.

### Possible Donors

Possible donors were identified from the registry data and included all patients born after 27 weeks of gestation, without diagnosis of necrotizing enterocolitis admitted to a neonatal intensive care unit in the greater Stockholm region [including the Karolinska University Hospital (Solna, Huddinge, and Danderyds hospital) and Södersjukhuset]. The region has a population of 2 million inhabitants, and the NICUs account for 22% of all hospitalizations of neonatal patients nationally^16^.

All possible donors’ medical charts were retrieved and reviewed for: medical history, weight, age, delivery, medication, mode of death (withdrawal of life-sustaining treatment—type, date, time), electroencephalogram (EEG) recordings, imaging, neurological examination, microbiological analyses, and biochemical analyses.

### Potential Donors

Assessment of potential donors was based on the data obtained from the medical charts. Possible donors with incomplete medical records, death occurring outside of the hospital, no active withdrawal of life-sustaining treatment, deceased in hospital unit outside of the NICU, and diagnose(s) not compatible with organ donation were excluded. Remaining patients were considered potential donors.

**Figure 1.**
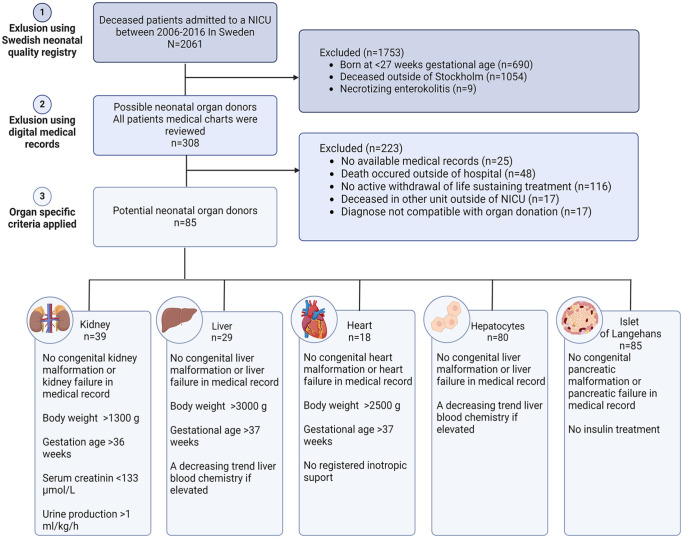
Inclusion of eligible neonatal donors in neonatal intensive care units within the central Sweden between 2006 and 2016.

Life-sustaining treatment was defined as treatment with ventilator, continuous positive airway pressure (CPAP), or treatment maintaining circulation using extra corporal membrane oxygenation (ECMO). Diagnoses not compatible with organ donation were trisomy 13 and 18, uncontrolled infection (defined as positive microbiological culture with increasing infection parameters), malignancies, and metabolic diseases. In addition, viral status for human immunodeficiency virus and hepatitis were assessed (Fig. 1).

Organ donation for respective organ was reviewed in all potential organ donors using organ specific criteria. Criteria used were based on previous publications and in collaboration with cardiac, kidney, and liver transplant surgeons (Fig. 1).

1. Kidney. Kidney organ specific criteria were based on previous publications on neonatal organ donation and kidney transplantation^17–19^. No congenital kidney malformation or kidney failure^17,19^. Body weight cut off >1,300 g was chosen as this is the lowest donor weight of successful transplanted neonatal kidneys, and gestational age >36 weeks^18^. Last denoted creatinine <1.5 mg/dl (133 µmol/l), and urine production >1 ml/kg/h^17,19^. In 10 cases no recent values of urine production could be noted. In these cases, only creatinine and body weight were used if the medical chart did not indicate any signs of kidney failure.
2. Liver. Liver specific criteria were based on previous publications on neonatal liver donation^19–22^. Body weight limit >3,000 g, gestational age >37 weeks^19–21^. As liver blood chemistry are commonly elevated following hemodynamic instability, no direct cut-off values for AST, ALT, INR, or bilirubin were used^23^. Instead, emphasis was placed on the medical history along with no congenital liver malformation, or signs of liver failure was denoted in the medical records^19^. However, if elevated ALT an/or AST the trend over time had to be showing decreasing transaminases.
3. Heart. Heart specific criteria were based on previous publications on neonatal heart donation^19,24,25^. Medical history indicating no congenital heart or major vessel malformation, and no signs of heart failure. No inotropic support^19,24^. Body weight >2,500 g and gestational age >37 weeks^24^.

Donation for cell isolation was deemed viable if following criteria were met:

1. Hepatocytes. Hepatocyte specific criteria were based on previous publications on neonatal hepatocyte donation^10,11^. Same criteria as applied for liver donation, although with no weight or gestational age restriction.
2. Islet of Langerhans. Neonatal islet cells are currently not used clinically and would be procured in a research setting, hence no cut off criteria for body weight or gestational age. No congenital malformation of the pancreas or pancreatic malignancies. No insulin treatment following birth.

### Potential DBD Donation

All potential donors were assessed as potential DCD or DBD donors; a person whose clinical condition is suspected to fulfill brain death criteria^15^. Based on current criteria for declaring neurological death used in the United Kingdom and in the United States in newborns, born after 37 weeks^26,27^. We considered all potential donors >37 weeks gestational age with a catastrophic brain injury [i.e. pathological EEG examination showing isoelectric or burst suppression pattern or radiological examination (MRI or ultrasound with signs of global ischemia or bleeding with mass effect)] in combination with a neurological examination showing lack of cranial nerve reflexes to fulfill criteria as a potential DBD donor.

### Statistics

Descriptive statistics were used, using median with interquartile range for continuous parameters and percentage and counts for categorical parameters. Total amount of potential organ donors was presented as a whole and for respective organ. Potential donation rate was presented as per million population (PMP) per year. PMP was calculated using the average population in the Stockholm County during 2006–2016 extracted from Statistics Sweden^28^.

All research was conducted in accordance with both the Declarations of Helsinki and Istanbul. Ethical approval for this study was obtained from the Swedish Ethical Review Authority.

### Ethics

Ethical approval for this study was obtained from the Swedish Ethical Review Authority: EPN 2017/1913-31/1.

## Results

### Study Cohort

A total of 2,061 neonates who eventually died were admitted to a NICU in Sweden between 2006 and 2016. The majority were male (55.5%) and the most common delivery method was emergency C-section (46.1%). Median gestational age was 32 + 4 (IQR: 12 + 4) with a median birth weight of 1,700 (IQR: 2,148) g. In total, 1,753 (85%) patients were excluded as possible donors based on registry data. A majority of patients, 51.1% (n = 1,054) were admitted to a NICU outside of the study area, 33.5% (n = 690) patients were severely premature born at a gestational age < 27 weeks, and 0.4% (n = 9) patients were diagnosed with necrotizing enterocolitis, remaining 15% (n = 308) were considered possible neonatal donors (Fig. 1, Table 1).

**Table 1.** Characteristics of Entire Cohort (N = 2,061), Possible (n = 308), and Potential (n = 85) Neonatal Organ Donors in Central Sweden Between 2006 and 2016.

	Entire cohort (N = 2,061)	Possible donors (n = 308)	Potential donors (n = 85)
	% (n = )		
Sex			
Female	44.4 (915)	47.4 (146)	49.4 (42)
Male	55.5 (1,143)	52.3 (161)	50.6 (43)
Ambiguous	0.1 (3)	0.3 (1)	-
Delivery method			
Vaginal	42.5 (875)	28.2 (87)	20 (17)
Emergency c-section	46.1 (950)	56.5 (174)	67.1 (57)
Elective c-section	7.2 (149)	11.4 (35)	5.9 (5)
Instrumental	3.8 (79)	3.9 (12)	7 (6)
Missing	0.4 (8)	-	-
CPR at time of birth			
Yes	76.5 (1,577)	76 (234)	89.4 (76)
No	20.5 (423)	22.4 (69)	9.4 (8)
Missing	3 (61)	1.6 (5)	1.2 (1)
Life-sustaining treatment withdrawn	-	38.3 (118)	100 (85)
Ventilator	-	31.8 (98)	83.5 (71)
ECMO	-	3.6 (11)	9.4 (8)
CPAP	-	2.9 (9)	7.1 (6)
Inotropic treatment	-	24.7 (76)	30.6(26)
Liver failure or malformation	-	3.2 (10)	3.5 (3)
Heart failure or malformation	-	29.5 (91)	49.4 (42)
Kidney failure or malformation		7.8 (24)	4.7 (4)
Active infection	-	14.9 (46)	2.6 (8)
Malignancy	-	2.6 (8)	0
	Median (IQR)		
Gestational age at birth (weeks + days)	32 + 4 (12 + 4)	35 + 6 (7 + 1)	35 + 1 (9 + 4)
Age at death (days)	3 (14)	8 (51)	3 (5.5)
Birth weight (g)	1,700 (2,148)	2,258 (1,420)	2,313 (1,903)
Weight at death (g)	-	2,540 (1,951)	2,355 (1,953)
Creatinine (µmol/l)	-	56.5 (61.2)	72 (67)
Diuresis (ml/kg/h)	-	3 (2.3)	3 (2.2)
ALT (µkat/l)	-	0.55 (1.6)	0.51 (1.5)
AST (µkat/l)	-	1.5 (2.8)	1.7 (3.1)
INR	-	1.6 (0.8)	1.6 (0.8)
HIV	-	0	0
HBV	-	-	0
HCV	-	-	0
Agonal time^a^ (min)	-	-	35 (64)

ALT—alanine aminotransferase. AST—aspartate aminotransferase. CPAP—continuous positive airway pressure. ECMO—Extra corporeal membrane oxygenation. HBV—hepatitis B virus. HCV—hepatitis C virus. HIV—human immunodeficiency virus.

a Agonal time, calculated from time of withdrawal of treatment until time of death occurred.

### Possible Donors

Compared with the entire cohort, possible donors were born later (gestational age 35 + 6 (IQR: 7 + 1) weeks) and consequentially heavier with a median birthweight of 2,258 (IQR: 1,420) g. Blood chemistry of interest among the possible donors were a median creatinine of 56.5 (IQR: 61.2) µmol/l, ALT 0.55 (IQR: 1.6) µkat/l, and AST 1.5 (IQR: 2.8) µkat/l. In total, 24.7% (n = 76) received inotropic support and 38.3% (n = 118) had life-sustaining treatment withdrawn. After retrieving and reviewing medical records of all possible donors, 72.4% (n = 223) were excluded. Reasons included: 8.1% (n = 25) patients with incomplete medical records, and in 15.6% (n = 48) patients death occurred outside of the hospital (24 deaths occurred in prehospital care, 13 in hospice care, 11 deaths occurred after discharge to another hospital outside of the county). In 37.7% (n = 116) of the patients, death occurred without active withdrawal of life-sustaining treatment (in 45 cases, death followed unsuccessful cardiopulmonary resuscitation). A total of 5.5% (n = 17) patients deceased in a unit outside of the NICU (two admitted to a pediatric unit, 15 to a pediatric intensive care unit). In addition, 5.5% (n = 17) patients were excluded due to a diagnosis not compatible with organ donation (seven neonates with trisomy 13 or 18, five uncontrolled infections, two malignancies, and two metabolic diseases). Remaining 85 patients were considered as potential organ donors (Fig. 1, Table 1).

### Potential Donors

Among the potential donors, sex distribution was 49% (n = 42) female and 51% (n = 43) male. Most common mode of delivery was emergency cesarean section which occurred in 67% (n = 57) of patients, followed by 20% (n = 17) instances of vaginal delivery, 7% (n = 6) instrumental deliveries, and 6% (n = 5) elective cesarean section. The type of life-sustaining treatment withdrawn within the active redirection of care was mechanical ventilation in 84% (n = 71) of the patients, in 9% (n = 8) ECMO, and for 7% (n = 6) of the patients CPAP treatment was ended. Inotropic support was administered to 31% (n = 26) of the potential donors, and median number of days on ventilator support was 3 (IQR: 4.2) days. Median birth weight was 2,313 (IQR: 1,903) g and gestational age was 35 + 1 (IQR: 9 + 4) weeks + days. Median age at death was 3 (IQR: 5.5) days (Table 1).

The most common cause of withdrawal of life-sustaining treatment was hypoxic ischemic encephalopathy (HIE), which was diagnosed in 47 (n = 40) of the 85 potential donors. In 36% (n = 31) of the cases underlying cause was respiratory insufficiency, 7% (n = 6) due to circulatory insufficiency, and 5% (n = 4) due to congenital disorder not compatible with life. This included fatal gracile bone dysplasia, major neurological malformation, and two patients with multiple malformations where genetic testing was negative for trisomy 13 and 18 (Table 2).

**Table 2.** Reasons of Active Withdrawal of Life-Sustaining Treatment in 85 Potential Neonatal Organ Donors in Central Sweden Between 2006 and 2016.

Cause of treatment withdrawal	
	N
**Hypoxic ischemic encephalopathy**	47 (40)
**Respiratory insufficiency**	36 (31)
Respiratory distress syndrome	11 (9)
Hypoplasia of the lungs	8 (7)
Persistent pulmonary hypertension	8 (7)
Congenital neurological disorder/malformation	7 (6)
Capillary alveolar dysplasia	1 (1)
Tracheal agenesis	1 (1)
**Circulatory insufficiency**	7 (6)
Hypertrophic cardiomyopathy	4 (3)
Hydrops fetalis	2 (2)
Malformation of major vessels	1 (1)
**Diagnose not compatible with life**	5 (4)
Multiple malformations^a^	2 (2)
Major neurological malformation	1 (1)
Fatal gracile bone dysplasia	1 (1)
**Intracranial hemorrhage/Intraventricular hemorrhage**	5 (4)

a Tested negative for trisomy 13 and 18.

After applying organ specific criteria for solid organs for each potential donor, a total of 39 pairs of kidneys, 29 livers, and 18 hearts could have been eligible for donation, corresponding to 1.9 PMP kidney donors, 1.4 PMP for liver, 0.9 PMP for heart. In total, 28% (n = 24) of the potential neonatal organ donors fulfilled organ specific criteria for two or more organ systems and could have been considered for multiple organ retrieval. In terms of potential donation of organs aimed at cell therapy, all 85 (4.1 PMP) met the criteria for donation of Langerhans islet cells, and 80 (3.8 PMP) met criteria for donation of hepatocytes (Table 3).

**Table 3.** Potential Neonatal Organ Donors in Neonatal Intensive Care Units in Central Sweden Between 2006 and 2016 and Distribution of Organs, for Donation After Circulatory Death and After Brain Death.

	Kidney	Liver	Heart	Hepatocyte	Islet
Potential DCD	39	29	18	80	85
PMP^a^	1.9	1.4	0.9	3.8	4.1
DBD	12	14	11	15	16
Weight at death (g)	3,004 (1,208)	3,456 (905)	3,415 (945)	2,319 (1,929)	2,355 (1,953)
Agonal time (min)^b^	38.5 (113)	40 (145)	65 (157)	33 (61)	35 (64)
Creatinine (µmol/l)	60.5 (45)	55 (66.5)	62 (66.8)	70.5 (66)	72 (67)
Diuresis (ml/kg/h)	3.7 (2.4)	3.0 (1.8)	3 (2.1)	3 (2.2)	3 (2.2)
Urea (mmol/l)	5.3 (3.9)	5.3 (4.2)	5.4 (4.15)	3 (4.4)	5.5 (4.1)
ALT (µkat/l)	0.5 (1.5)	0.5 (1.3)	0.6 (2.5)	0.4 (1.3)	0.51 (1.5)
AST (µkat/l)	1.4 (2.1)	1.9 (2.2)	1.9 (2.0)	1.7 (2.6)	1.7 (3.1)
Bilirubin (µmol/l)	37 (44.8)	36 (89)	37 (89.3)	62 (112)	52.5 (108)
INR	1.5 (0.5)	1.4 (0.5)	1.5 (0.5)	1.6 (0.8)	1.6 (0.8)

ALT—alanine aminotransferase. AST—aspartate aminotransferase. DBD—donation after brain death. DCD—donation after circulatory death. PMP—Per million population per year.

Source: Statistics Sweden.

a Based on average population in Stockholm County 2006–2016 (2,091,134 inhabitants).

b Agonal time, calculated from time of withdrawal of treatment until time of death occurred.

No case of maternal HIV and two cases of maternal hepatitis B were recorded. In one of the cases the mother had a previous infection, with negative HBV DNA quantification in the neonate. In the other case, the mother suffered from an ongoing hepatitis B infection. Repeated DNA quantifications were negative in the neonate, with negative HBsAg (Table 1).

Among the 39 potential kidney donors, median weight was 3,004 (IQR: 1,208) g, creatinine 60.5 (IQR: 45) µmol/l, diuresis of 3.7 (IQR: 2.4) ml/kg/h, and urea 5.3 (IQR: 3.9) mmol/l. In two patients, no values were denoted for creatinine or urine production, but the medical charts did not indicate any signs of kidney failure. In 10 patients, there were no information on diuresis; however, creatinine was satisfactory. Agonal time noted in medical records was 38.5 (IQR: 113) min (Table 3).

Among the 29 potential liver donors, median weight was 3,456 (IQR: 905) g. Blood chemistry showed ALT 0.53 (IQR: 1.3) µkat/l, AST 1.9 (IQR: 2.2) µkat/l, bilirubin of 36 (IQR: 89) µmol/l, and INR 1.4 (IQR: 0.5). In two patients, there were no liver samples recorded, the medical charts did not indicate any liver failure. Recorded agonal time was 40 (IQR: 145) min (Table 3).

Median weight for potential heart donors was 3,415 (IQR: 945) g. Of the 18 potential heart donors, 55% (n = 10) had a cardiac ultrasound noted in the medical charts, which showed normal anatomy and contractility. Four patients had patent ductus arteriosus, which was not regarded as a contraindication for heart donation. Agonal time recorded was 65 (IQR: 157) min (Table 3).

### Potential DBD Donors

Among the 85 potential donors, medical charts showed that 18.8% (n = 16) had catastrophic neurological injury in combination with a neurological examination without response to cranial nerve stimulus. Cause of death was HIE in 15 patients and one patient was born with severe neurological malformations leading to respiratory insufficiency (Table 3).

## Discussion

Our results indicate that there is an unused potential source of organs for transplantation in NICUs in Sweden. Of a total of 308 possible neonatal donors in the Stockholm area during the study period, we found 85 potential neonatal organ donors in neonatal intensive care units. In theory, a neonatal donation program could potentially contribute up to 0.9 to 1.9 PMP additional deceased organ donors, depending on organ. Comparatively, in 2022 there were a total of 206 utilized donors in Sweden with an overall donation rate of 19.6 PMP, (34.8 PMP for kidney 15.8 for liver, 5.1 PMP for heart)^29^. However, our study does not consider consent rate, which would likely reduce the number of actual neonatal donors. Sweden has among the highest consent rates toward organ donation globally^30^. Although this has not been explored within a neonatal context, in a previous study evaluating hepatocytes procured from neonatal donors, consent rate for study inclusion was high^11^.

Similar studies investigating potential DCD donors in NICUS, without applying organ specific criteria, found a potential ranging from 6% to 40% of deaths within a NICU^7^. When applying organ specific criteria potential donation rates have varied between 9% and 33% for kidney, 2% to 5% for heart, and 7% for livers^7^. Similarly, we found a rate of 12.6% for kidneys, 9.4% for liver, and 5.8% for heart. Although results between these studies can be difficult to compare due to differences in study methodology, results provide an indication of the potential of neonatal organ donation which is in line with what has been observed elsewhere.

A study by Charles et al. evaluated DBD in neonates, eligibility was assessed by evaluating EEG, brain stem reflexes and pupil response to light. During 5 years, 84 patients between 37 weeks up to 2 months died, of which 13% were deemed eligible for DBD^31^. In comparison we found 5.2% of cases where DBD could have been plausible, although requiring further evaluation. Currently, direct neurological criteria for brain death are not utilized in neonates in Sweden, hence none of the patients included in the study population underwent a formal brain death evaluation with apnea testing. Although DBD is possible in neonates, total brain infarction is uncommon due to patent sutures allowing for accommodation of increased intracranial pressure. A more likely avenue for organ donation in this patient group is DCD. Although there are no regulatory limitations precluding neonates from becoming DCD organ donors in Sweden, it is currently not implemented. According to present Swedish directives, death cannot be declared in neonates until 20 min after withdrawal of life-sustaining treatment, which could extend the no touch period to longer than 5 min (which is applied in pediatric and adult donors), thereby potentially extending the functional warm ischemia time^32^.

Outcomes following transplantation with organs procured from DCD donors have historically been inferior compared with DBD, mainly due to damage caused during warm ischemia^33^. However, advancements in *in situ* and *ex situ* machine perfusion provides new strategies in organ procurement and preservation. *Ex situ* normothermic preservation of hearts provides additional time for transportation compared with static cold storage and allows for further assessment after procurement, moreover it seems to mitigate additional ischemic damage which occurs during DCD procurement providing similar outcomes to DBD^34^. *In situ* perfusion with the utilization of thoraco-abdominal normothermic regional perfusion, allows for organ evaluation prior to procurement in DCD donors, and has shown to improve postoperative outcomes for DCD liver and heart transplantation comparable with DBD^33,35^. Moreover, although speculative, it is possible that neonatal organs may tolerate a longer period of warm ischemia than the limits currently applied clinically. Previous animal studies suggest that neonatal cardiomyocytes may have a higher ischemic tolerance compared with adults^36,37^. Moreover, in a study on human neonatal hepatocytes, high hepatocyte viability was found despite long periods of warm ischemia^11^.

Granted that there is a potential of neonatal donation, other factors need to be taken into consideration. In terms of transplantation of livers procured from neonatal donors only two series have been published, while transplanted livers were well functioning, both studies reported high rates of artery thrombosis^20,21^. Wijetunga et al. published the largest series of en bloc kidney transplantations from small donors (<5 kg, including neonatal donors) showing excellent function. However, the authors conclude that there may be an increased risk of vessel thrombosis^18^. Hence, further investigations into modified anticoagulative protocols postoperatively may be needed within this donor group. Another important aspect is the size-matching, although neonatal kidneys can be utilized for adult patients, liver, and hearts require size matching, consequently timing between a neonatal donor and a suitable recipient is required. Boucek et al.^25^ published the utilization of neonatal hearts procured in a DCD setting with excellent results, yet the authors describe a challenge in timing and logistics and therefore utilized a collocating protocol of the donor and recipient. The authors experienced that the likelihood of having a neonatal donor concurrently as a suitable recipient was small. This effect could be especially pronounced in a low populated country, emphasizing the importance of organ sharing networks and centralization. Moreover, recent advancements in organ preservation can extend ischemic and transportation times. A recent publication illustrated the enormous advantage and potential for expansion of cardiac transplantation due to machine perfusion^38^. Beckerman et al. utilized normothermic regional perfusion when procuring a heart from a 2-month-old DCD donor after 63 min of agonal time. Following reanimation of the heart, it was further assessed *in situ* using ultrasound, whereafter it was procured and successfully transplanted into a 6-month-old recipient following 198 min of cold ischemia time.

Introducing neonatal organ donation does not only have the possibility of aiding another individual suffering from end stage organ disease but could also bring a feeling of meaningfulness to the bereaved parents. A study by Hoover et al.^39^ found that parents to pediatric organ donors experienced the situation as a meaningful contribution and positively influenced the grieving process. Conversely, parents have reported negative experiences regarding the viability of the organs or when learning about the recipient. Examples include parents hoping for the possibility of donation, but due to prolonged warm ischemia time donation was inhibited^39^. Or parents experiencing disappointment that the organs did not go to another child but rather to an older recipient^39^. These aspects highlight the importance of transparency and public education when introducing a neonatal organ donation program.

As the need for organs increases there is an increasing utilization of marginal donors, in particular older donors^40^. Previous studies have shown that organs from older donors may have increased rates of acute rejections due to altered immunogenicity and to develop^41^. In addition, research in animal models indicates that organs from older donors promote cellular senescence in recipients^42^. Possibly, utilizing organs neonatal donors could prove beneficial in reducing acute rejections, improve cellular healing, and organ longevity.

The study at hand is limited by the inherent biases associated with retrospective studies, (completeness of data and unobserved confounders). Moreover, our study does not include a limit on agonal time nor on warm ischemia times which indeed are relevant in DCD donation and is included in the definition of an eligible donors^15^. The reasoning was that following withdrawal of life-sustaining treatment within neonatal care in Sweden, there is no active monitoring of the patient until time of death, as to not disrupt the grieving process of the parents. In addition to high missing data (11%) recorded agonal time in medical records is likely unreliable. When moving forward in the donation process from potential to eligible donors there will be a reduction due to warm ischemia limits. The agonal times recorded for kidney and liver (39 and 40 min, respectively) are within the plausibility of acceptance. Conversely, recorded agonal time for potential heart donors was higher (65 min), possibly owing to the selection criteria for potential heart donors resulting in neonates with healthy strong hearts, resulting in longer time from withdrawal of life-sustaining treatment until declaration of death. Limits for warm ischemia time for DCD donors is not unequivocally clear, with machine perfusion allowing for extended ischemia times not only for hearts but also abdominal organs^33,43,44^. Furthermore, conversion of eligible to actual donors includes consent, which will result in a further reduction. Conversely, the study is strengthened by the completeness and coverage of SNQ, and that complete available medical records of all the 308 neonatal deaths have been validated^14^.

As the supply of organs available for transplantation is in an imbalance with the increasing demand, new ways of expanding the donor pool are constantly sought after. Currently neonatal organ donation from neither DBD nor DCD is not considered in Sweden. However, it is implemented in other countries, with good results. Implementing a donor program in Sweden for neonatal donors could significantly increase the donor pool, also enabling size matched transplantation for younger recipients. Advancements within organ preservation using *in situ* and *ex situ* perfusion provides novel strategies with the possibility of extending transportation as well as ischemia times. In addition to possibly saving lives of young recipients in need of an organ transplantation, a neonatal donation program could enable further research on cell therapies such as transplantation of hepatocytes and islet of Langerhans.
